# Light during embryonic development modulates patterns of lateralization strongly and similarly in both zebrafish and chick

**DOI:** 10.1098/rstb.2008.0241

**Published:** 2008-12-04

**Authors:** R.J. Andrew, D. Osorio, S. Budaev

**Affiliations:** School of Life sciences, Centre for Biology and Environmental Sciences, University of SussexBrighton BN1 9QG, UK

**Keywords:** lateralization, light, zebrafish

## Abstract

Some aspects of lateralization are widespread. This is clear for the association between left-eye (LE) use and readiness to respond intensely to releasing stimuli presented by others, which has been found in representatives of all major groups of tetrapods and in fishes. In the chick, this behavioural asymmetry is linked developmentally to greater ability to sustain response against distracting stimuli with right-eye (RE) use, in that both reverse with the reversal of the normal RE exposure to light. In the zebrafish, the same two asymmetries (normally) have similar associations with the LE and the RE, and both also reverse together (owing to epithalamic reversal). Here, we show that light exposure early in development is needed in zebrafish to generate both asymmetries. Dark development largely abolishes both the enhanced abilities, confirming their linkage. Resemblance to the chick is increased by the survival in the chick, after dark development, of higher ability to assess familiarity of complex stimuli when using the LE. A somewhat similar ability survives in dark-developed zebrafish. Here, LE use causes lesser reliance on a single recent experience than on longer term past experience in the assessment of novelty. Such resemblances between a fish and a bird suggest that we should look not only for resemblances between different groups of vertebrates in the most common overall pattern of lateralization, but also for possible resemblances in the nature of inter-individual variation and in the way in which it is generated during development.

## 1. Introduction

Some features of lateralization are widespread among vertebrates (Andrew & Rogers 2002). The left-eye system (LES) has an advantage in the analysis of topography and position (chick: Rashid & Andrew 1989; Tommasi *et al*. 1997; marsh tit: Clayton & Krebs 1994; rat: Bianki 1988; human: Kosslyn *et al*. 1992). Furthermore, the LES is more likely to respond to various releasers. This is true of attack and sexual behaviour, when it is the left eye (LE) that sees the stimulus rather than the right (e.g. toad: Robins *et al*. 1998; lizard: Deckel 1998; chick: Rogers *et al*. 1985; baboon: Casperd & Dunbar 1996).

By contrast, the right-eye system (RES, left hemisphere) use is associated with a response to an identified target such as a prey item (zebrafish: Miklósi & Andrew 1999; toad: Vallortigara *et al*. 1998; chick: Andrew *et al*. 2000). In the rat, the left hemisphere is especially involved in a rapid choice of the correct response (Bianki 1988).

Now that it is clear that cerebral lateralization is very widespread among vertebrates, the next objective must be to understand the extent to which it varies between individuals and between species. A major obstacle is that the observed behavioural asymmetries may be affected by factors that are not themselves integral to lateralized brain mechanisms. Moreover, such factors may affect different behavioural asymmetries differently. It is very clear in fishes, for example, that motivational differences, which change the significance of test stimuli, can also change the observed behavioural asymmetries; thus, the evocation of sexual motivation can bring different brain mechanisms to bear, generating behavioural asymmetries of a different kind (Bisazza *et al*. 1998). A promising simplification is to study the effects on well-established behavioural asymmetries of known changes in brain lateralization.

Here, we present evidence for unexpected resemblances between the domestic chick and zebrafish in behavioural lateralization. In both, the same two asymmetries are tightly linked in development, in that both reverse their allocation together: when one shifts from association with right-eye (RE) use to association with the LE, the other shifts in the opposite direction.

In the chick, the first asymmetry (visual control of response, VCR) is the use of the RE to select and sustain an approach to a target (Andrew *et al*. 2000), and to sustain selection of targets rather than distractors (Rogers & Anson 1979); this bias is present also in the zebrafish (Miklósi & Andrew 1999). The second is the readier evocation of responses when appropriate and highly effective stimuli are perceived with the LE rather than with the RE. The examples so far described chiefly relate to the releasers of responses such as attack and sexual behaviour (chick: Rogers *et al*. 1985; many groups of vertebrates: reviewed in Vallortigara & Bisazza 2002). The widespread use of the LE when a fish views its own reflection (Sovrano *et al*. 2000) suggests that the releasers of social responses are also more effective when seen with the LE. This use of the LE is also present in the zebrafish (Andrew *et al*. in press).

Here, we add an additional class of releasers for the zebrafish: we used a simple pattern providing visual heterogeneity, such as might serve to identify a potential refuge, and showed that there was an asymmetry of response according to which eye saw it. A convenient overall term for such a feature of behaviour is a ready response to releasers (RRR).

In both species, VCR and RRR are linked in development. In the chick, they both reverse their allocation as a result of experimental reversal of the normal asymmetric exposure of the eyes late in development (Deng & Rogers 1997). The RE normally faces outwards, and so receives light entering through the shell. If the LE is exposed to light instead, then VCR and RRR shift in the opposite directions (Rogers 1990). In the zebrafish, the reversal of the habenular asymmetry results in exactly the same shifts as in VCR and RRR (Barth *et al*. 2005). Eye use in mirror tests reverses, and the LE is used instead of the RE in an approach to a target.

Developmental effects of light in the zebrafish during development have already been demonstrated. These include the loss of bias to LE use in the mirror test when light is absent during development (Andrew *et al*. in press), and elevation of boldness in fry exposed to light (Budaev & Andrew in press). A major component of the boldness syndrome was elevated locomotion in a strange environment, which correlated with the degree of approach to a predator model. Both these features of behaviour would be well explained by an enhanced ability to sustain a response (i.e. elevated VCR), once initiated, which is present in light- but not dark-developed zebrafish.

In the chick, the absence of light late in development minimizes (or perhaps abolishes) both behavioural asymmetries (Rogers 1982; Zappia & Rogers 1983). Here, we provide similar evidence for zebrafish. This is important because it suggests that light is involved in the generation of the specific brain asymmetry (or asymmetries) that generates VCR and RRR, rather than in some way reversing the direction of brain lateralization as a whole. This conclusion is reinforced in dark-developed chicks by the presence of some clear differences between behaviour shown with the LE or the RE in use (Mascetti & Vallortigara 2001; Deng & Rogers 2002; Chiandetti *et al*. 2005). It is particularly important that higher interest in novelty when the LE is in use is present in dark chicks (Rogers *et al*. 2007).

## 2. Material and methods

Breeding zebrafish came from a pet shop (Brighton, UK). They were maintained in aquaria (300×155×155 mm) at 28°C on a 14 L : 10 D cycle, and fed daily with dry zebrafish pellets (ZM-400; ZM Ltd, Winchester, England, UK). We used fry from four different batches coming from the spawning of one female with two males. We did not note pronounced differences in behaviour or other characteristics between these batches. Approximately 3 hours after fertilization, the eggs were removed from the parental aquarium and transferred to white plastic boxes (140×80×50 mm). The eggs and the larvae (‘fry’) were maintained in these boxes in groups of approximately 15–20. For protection against fungi, a few drops of methylene blue solution were added to each box.

Approximately 6 hours after fertilization, the eggs were divided into two experimental groups, held in boxes in separate aquaria at 28°C. The first group was maintained under the normal 14 L : 10 D cycle. The second group developed in darkness (<0.01 lux measured with Extech EasyView EA30 digital light meter). The eggs and fry of the dark group were taken to light only for a short time (<2 min each time) every second day for maintenance, inspection and cleaning.

The dark group was returned to the normal 14 L : 10 D cycle in the evening of the day 6 post fertilization. On the 8th day post-fertilization (DPF), both the light and dark groups were further divided into two treatments. The first treatment (EXPER) had home-box experience of the vertical black stripe stimulus used in subsequent behavioural experiments, whereas the second (NO EXPER) had no such experience. The black stripe was inserted in the middle of the longer walls of the white maintenance boxes of the EXPER group, whereas NO EXPER fry were maintained in empty boxes. The behavioural experiments were conducted on 11 DPF, so that the EXPER group had 3 days experience of the black stripes in their home boxes. The fry were not fed prior to the tests.

**Figure 1:**
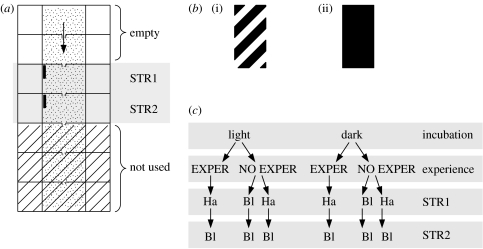
Scheme of the experiments. (*a*) An outline of the swim-way used in the experiments with the sequence of tests (indicated by an arrow, from top down); (*b*) the stimuli used in the STR1 and STR2 tests: (i) hatched and (ii) black stripes; and (*c*) an outline of the groups used in the study. Ha, hatched stripe; Bl, black stripe.

All tests used a white plastic swim-way (320×125 mm) with seven compartments (50×40 mm, from which we used only four) filled with water to a depth of 25 mm (figure 1*a*). All adjacent compartments were connected by vertical 5 mm slits in the middle of the connecting walls. Two plastic bars were attached at each side of the slit to create a 9 mm long corridor (figure 1*b*). Each compartment contained two lamps mounted above the left and right sides, which could not be directly seen by the fry. The lighting of each compartment was controlled by switches and a rheostat. A small video camera could be slid along a glass sheet covering all the compartments and monitoring each in turn. The whole apparatus was covered by black cloth to exclude light from other sources. The individuals were tested only once and water in the experimental swim-way was changed after each fry. Full details are given by Watkins *et al*. (2004) and Budaev & Andrew (in press).

The testing procedure is schematized in figure 1*a*. At test each fry was sucked into a large pipette (entrance diameter 6 mm), together with an adequate amount of water, and released gently into the first compartment of the swim-way, which was lit by the lamps. All other compartments were darkened. The fry was left undisturbed for 4 min. Subsequently, the light in the test compartment was slowly dimmed to darkness over 20 s. The video camera was immediately shifted to monitor the second compartment. Illumination was then similarly raised in this second compartment. The fry entered this novel compartment under positive phototaxis.

After entering the second compartment, the fry was left to explore it for a further 1 min. Thereafter, the same sequence of changes in lighting was used to attract the fry into subsequent compartments (figure 1*b*). The third and fourth compartments had a large vertical stripe at the closest end of the entry wall, which was on the left or the right, keeping the same position for the first and second stripe tests (STR1 and STR2). The stripe was placed so that it could be seen monocularly before entry, but only when the fry had arrived at the end of the short between-compartment corridor. Two stripes were used: a hatched stripe made up of alternating black and white lines, 5 mm wide and angled at 45°, and a uniformly black stripe of the same size (figure 1*b*). After entry, the fry was left to explore the compartment and stripe for 1 min. Three designs were used (figure 1*c*). The EXPER fry saw a hatched stripe in STR1 and a black stripe in STR2, while NO EXPER saw either a hatched stripe, followed by a black, or a black in both the tests. One reason for this design was that it was already known (Andrew *et al*. in press) that RE use tended to be dominated by immediately prior experience, whereas LE use was more affected by long-term experience.

The behaviour of the fry in both the stripe tests was video recorded and analysed using an on-screen measurement software. We recorded distances between the fry and the stripe every 10 s. In the following analysis, we use the minimum distance achieved during the test between the fry and the stripe. This allowed us to measure whether the fry responded differently to the stimuli presented to the LE or the RE. The sample sizes within each experimental combination were slightly different (*n*=4–7) because of fry availability. The total number of fry was 42. We used ANOVAs and randomization tests for statistical comparisons (R software package; *p*-values are two-tailed).

## 3. Results and discussion

### (a) Overall analyses

**Figure 2:**
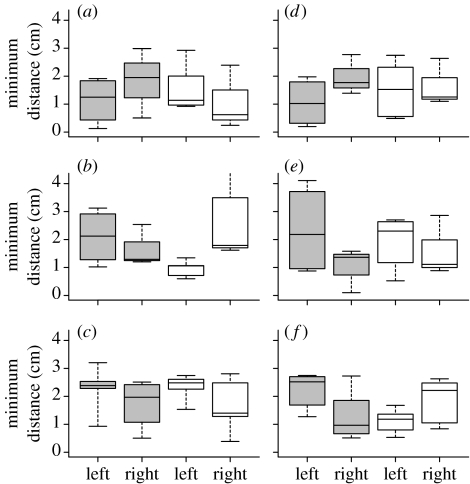
The minimum distance to the stimulus in the different experimental groups. (*a*–*c*) The first stripe test (STR1) and (*d*–*f*) the second stripe test (STR2). The plots include experience, condition and the type of the stimulus (e.g. EXPER, hatched for experienced fry in test with the hatched stripe): (*a*) EXPER, hatched; (*b*) NO EXPER, hatched; (*c*) NO EXPER, black; (*d*) EXPER, black; (*e*) NO EXPER, black; (*f*) NO EXPER, black. Dark fry, grey boxes; light fry, white boxes. Median, quartiles (25–75%) and extremes are shown.

Light and dark fry showed different left/right patterns (figure 2). Overall, the experimental groups exhibited significant differences in their responses in the STR1 test (experience, EXPER or NO EXPER; side, stripe on the left or the right; development, light or dark: experience×side×development *F*_1,22_=5.194, *p*=0.033). This was partly due to the different responses of the NO EXPER fry (stimulus, hatched or black; stimulus×side: *F*_1,34_=7.037, *p*=0.012; side×development: *F*_1,34_=4.941, *p*=0.033). There was no overall significant differences between the groups in the second stripe (STR2) test, but the two NO EXPER STR2 groups still showed significant differences (side×development: *F*_1,35_=6.676, *p*=0.014).

### (b) Light fry

Within the NO EXPER groups, the left/light fry showed a close approach to the hatched but not to the black stripe, while the right/light fry showed if anything the reverse (figure 2). In the STR1 test for the light fry, there was, as a result, a significant interaction in the NO EXPER condition for stimulus×side (*F*_1,17_=10.459, *p*=0.005). The close approach by the left/light fry to hatched in the STR1 test was unaffected by EXPER, when STR1 hatched tests were compared between EXPER and NO EXPER (figure 2). The right/light fry, by contrast, showed a striking close approach in the EXPER condition, when compared with NO EXPER. As a result, the interaction for side×experience, in the hatched tests, was suggestive (*F*_1,12_=4.64, *p*=0.052), entirely due to the change in the right/light groups.

The absence of a close approach in the NO EXPER condition was accompanied by pronounced inter-individual differences in the pattern of the latency distribution: some individuals showed very long latencies, although most came out quickly as usual. This wide spread was completely absent in the EXPER condition (and in left/light in both conditions), suggesting that some right/light individuals had difficulty in assessing the hatched stripe when seen in the absence of any comparable experience. The difference in variances between the EXPER and NO EXPER conditions in right/light was significant (Ansari–Bradley (AB) test, *n*=28, AB=132, *p*=0.0129). The very different behaviour in the EXPER condition is perhaps due to a combination of readiness to accept before the emergence a crude match with the record from home-tank experience, coupled with a high ability to sustain an approach to examine further (see §4).

The special response of the left/light fry to hatched stripes was absent in left/dark (figure 2). A comparison of the response to the black or hatched stripes in STR1 NO EXPER in the left tests produced a significant interaction (stimulus×development: *F*_1,19_=5.145, *p*=0.035), owing to the anomalous approach by left/light.

The special response of light fry to the properties of hatched when seen by the LE is probably one aspect of greater responsiveness of the LES to valent stimuli: in this case, visual heterogeneity of the environment, such as might be presented by potential refuges or landmarks (see §4).

### (c) Dark fry

An important issue here is whether there are any left/right differences in the dark fry. The left/dark and right/dark fry were affected differently by home-tank experience (figure 2). A comparison of the EXPER group with both NO EXPER groups in the STR2 test showed a significant interaction (experience×side: *F*_1,24_=5.739, *p*=0.025). The experience of black in the home tank caused left/dark to approach, presumably because the stripe was assessed to be a familiar refuge or landmark. In the absence of home-tank experience, black was not approached. The right/dark fry showed a quite different pattern: they approached closest when they had had one previous experience of the same stripe (i.e. in the NO EXPER group with black in the first and second emergence) and least when they had home-tank experience of black.

These results are best explained by supposing that, in the dark fry, the LES relies to a marked degree on the use of established traces in assessing the stripes. The left/dark fry were unaffected by a single experience of either a black or hatched pattern in a second encounter with a stripe: whether there had been home-tank experience or not, the STR1 and STR2 responses were almost identical. If the black stripes had been present in the home tank, then the stripes (whether black or hatched) were approached, presumably because they were judged (relatively) familiar and interesting. Even in the case of the black stripes, the stimulus was a familiar object in a strange place. If no stripe had been seen in the home tank, then avoidance was persistent.

The right/dark groups clearly differ. In the NO EXPER condition, a prior brief exposure to black caused approach to appear in STR2, quite unlike the absence of change in left/dark. By contrast, after home-tank experience of black, black is not approached in STR2, (unlike both NO EXPER groups). This may represent the assessment as a familiar landmark or obstacle, which should be ignored; in any event, EXPER affects the right/dark fry, but quite differently from the left/dark fry.

It is impossible to tell whether a comparable left/right difference is present in the light groups, because of the special response of left/light to hatched. This does not apply to the NO EXPER condition, with black in both STR1 and STR2. Here, there was a change in the pattern of response owing to the first exposure (figure 2), with left/light increasing approach in STR2 and right/light avoiding more (side×test: *F*_3,47_=3.417, *p*=0.025, repeated-measures ANOVA). This was chiefly due to the change in left/light (two-sample permutation test: left/light *n*=14, *T*=1827, *p*=0.002; right/light *n*=13, *T*=1081, *p*=0.677).

Here, the STR1 exposure changes behaviour in STR2 in both left/light and right/light, but not in the corresponding dark groups. Only a tentative interpretation is possible and the effects require replication. The marked shift to approach shown in STR2 (black) by left/light may represent treating black as a refuge, once it is familiar. This would probably be advantageous: taking up a position against a large dark fixed object would make the fry less conspicuous. The avoidance shown by right/light in STR2 may reflect a decision taken in the first emergence that the black stripe is an obstacle, which is strongly sustained in the second emergence. Another odd result, which also requires further work, is the fact that the right/light groups differ suggestively between EXPER and NO EXPER for STR1 hatched. Hatched is avoided in the NO EXPER condition, but approached very close in the EXPER condition (side×experience: *F*_1,12_=4.641, *p*=0.052). Here, home-tank experience may allow a judgement of sufficient novelty as to cause a close approach in investigation, which is then sustained by high VCR.

## 4. General discussion

The abilities that in both a fish and a bird depend on the effects of light during development (VCR and RRR) are linked in the zebrafish to the habenular asymmetry, in that both reverse in association with the reversal of the habenular asymmetry (Barth *et al*. 2005). The normal asymmetry of the zebrafish habenula is for lateral habenula enlargement on the left and medial enlargement on the right (Aizawa *et al*. 2005). Outflow is strikingly separate, with the left lateral habenula predominantly supplying the dorsal division of the main relay nucleus (the interpeduncular nucleus, IPN), and the right medial habenula the ventral IPN. There is a striking resemblance between the functions of the lateral habenulae in zebrafish and mammals: in rats during a targeting and pursuit task, firing in the lateral habenular units correlates with targeting head movements (Sharp *et al*. 2006). The use of the RE in the selection and pursuit of targets by zebrafish (Barth *et al*. 2005) is clearly comparable, as is the ability to sustain choice by inhibiting pecks at irrelevant stimuli shown by chicks when using the RE (Deng & Rogers 1997, 2000). The lateral habenulae are also concerned in mammals, with the inhibition of response to the stimuli that predict the absence of reward in a task where reward is expected (Matsumoto & Hikosaka 2007), and with the inhibition of premature responses (Lecourtier & Kelly 2005). These two functions can be reconciled if it is supposed that one of the ways in which the lateral habenulae sustain an ongoing response is to inhibit a response to other stimuli, which are potentially powerful in evoking a response.

The medial habenulae are involved in reward in mammals (as shown by the effects on self-stimulation for morphine; Taraschenko *et al*. 2007). The zebrafish findings suggest an extension of this function to the promotion of response to any stimulus that is intrinsically powerful in evoking a response. The LE viewing of a mirror reflection is shown only by the light-developed fry (Andrew *et al*. in press). Such persistent bias strongly suggests that a response is here being driven by especial interest of the LES in the motivating properties of social fellows. The dark fry do not lack interest in their reflection. They show persistent viewing, but with alternating long periods of LE or RE use, allowing both eye systems an opportunity to assess. It is thus chiefly the asymmetry that is lacking.

Here, we have shown that the left/light, but not the left/dark, fry respond strongly (and innately) to properties, which are likely to be associated with potential refuges. The dissected character of the stimulus matches the properties of vegetation or debris. Small fishes are often attracted to visually heterogeneous patterns such as alternating stripes in a novel environment and begin to feed earlier in their proximity (Mikheev *et al*. 1997). Also, the presence of visual heterogeneity may reduce the fear response of fishes to the predator's odour (Afonina *et al*. 2005). Approaching such potentially camouflaging heterogeneous patterns should therefore be adaptive for small fry. On the other hand, fry should avoid visually homogeneous areas where they would be more conspicuous to predators. Taken together, the evidence suggests that after light, but not dark, development, the LES has heightened interest in, and response to, motivating stimuli. It will be interesting to see whether this extends to conditioned stimuli that were originally neutral, but have become very valent.

### (a) Asymmetry present in the dark fry

Zebrafish fry show some behavioural asymmetry after dark development. Here, we have shown that the left/dark fry are strongly affected by long-term experience of stripes in the home tank, but (unlike right/dark) hardly at all by the nature of the stripe seen in the first emergence.

Such an asymmetry is likely to interact with the assessment of novelty and complexity. The preferential and sustained use of established long-term records would promote such assessment. Greater use of recently elaborated records (such as is present in the right/dark, and perhaps in the right/light fry) would oppose accurate evaluation of identity, both in the long run by making difficult use of records that allows many experiences to be taken into account, and in the short term by making records based on current experience, the main determinants of judgements of familiarity. However, it would, at the same time, promote continuing choice of a particular stimulus or type of stimulus.

Dark-developed chicks show a greater interest in and ability in assessing novelty when using the LES (Deng & Rogers 2002; Andrew *et al*. 2004; Rogers *et al*. 2007). This is thus another probable resemblance between a fish and a bird.

### (b) Functions of lateralization

A number of functions have been suggested, which may all be to some extent true. The existence of (basically) similar suites of structures on the right and the left probably does ‘increase capacity’ when the two are independently searching different areas of space. It is also likely that some functions require neural organizations incompatible with other functions, if they are to be well performed. Establishing the identity of a complex stimulus with past experience requires extensive use of a detailed record, in which the properties of the record determine the analysis. The behaviour of the left/dark fry, which is reported here, may provide an example of a high degree of such use. This is quite different, for example, from the use of a particular cue to select possible targets efficiently and quickly. Here, the choice of eye with which to view allows appropriate abilities to be brought to bear.

However, it is likely that the determination of the sort of behaviour that is likely to be evoked is an additional important function. The sort of variation that is important here is whether initiated responses are sustained against distraction and danger, or whether much time is spent investigating slight change. The effects on such suites of behaviour might act over relatively short intervals: motivational factors such as being frightened are an obvious example. Alternatively, the effects might act over longer periods or a complete lifetime. Here, long-acting physiological conditions such as levels of sex hormones, or genetic and environmental effects during development (such as are considered here) are likely causes.

Variations in boldness are a revealing example since their consequences under natural conditions have been extensively studied in great tits. Drent *et al*. (2003) summarized the properties of the two extreme personality types. Active (bold) individuals take rapid decisions, establish routines based on past success and are aggressive to fellows. Passive (shy) individuals are cautious, responsive to external stimuli rather than sustaining routines (so that exploration is thorough) and less aggressive.

The light fry are bold and the dark fry are shy, as measured by a ‘shyness’ construct, derived from the tests in the same apparatus as that used in the present study (Budaev & Andrew in press). The construct was derived from levels of locomotion after entry to compartments, and the degree of approach to a crude model of the front view of a larger fish, measures which showed a positive correlation. Boldness in the light fry is likely to be due to the developmental effects of light on VCR, making it easier for locomotion to be initiated and sustained in a novel environment, including approach to a potentially dangerous stimulus. The effects on the development of an environmental factor, which could act in the field, offers a source of inter-individual variation, which may be more resistant than genetic control to fixation of one or other extreme pattern of behaviour, to fixation within a population by strong selective pressures.

## 5. Conclusion

Past speculation (e.g. Andrew 2002) has raised the possibility that a single pattern of lateralization is at least widely characteristic of vertebrates. It is now necessary to change this hypothesis by adding that there is commonly inter-individual variation in some aspects of this pattern, which, however, may itself be relatively standard. The resemblances set out here between a fish and a bird suggest a specific hypothesis. When there is a marked behavioural asymmetry in VCR and RRR in an individual, LE use (right brain control) will go with elevated RRR, and RE use (left brain control) with high ability to sustain an initiated response. Inter-individual variation will involve shifts between behavioural asymmetry and near symmetry in these behavioural patterns. At the same time, other aspects of lateralization, in particular the LES assessment of novelty or identity, will not vary (except as an indirect consequence of change in VCR and RRR).
